# Supplementary material for: Adult Inception of Ketogenic Diet Therapy Increases Sleep during the Dark Cycle in C57BL/6J Wild Type and Fragile X Mice
Source: Int J Mol Sci. 2024 Jun 18;25(12):6679. doi: 10.3390/ijms25126679 (PMC11203515; doi:10.3390/ijms25126679)
Supplement: Supplementary file 1 [file ijms-25-06679-s001.zip › ijms-3062545-supplementary.pdf]

International Journal of *Molecular Sciences*

MDPI

Supplementary Data

Adult Inception of Ketogenic Diet Therapy Increases Sleep during the Dark Cycle in C57BL/6J Wild Type and Fragile X Mice

Pamela R. Westmark [1,†], Timothy J. Swietlik [1,†], Ethan Runde [1], Brian Corsiga [1], Rachel Nissan [1], Brynne Boeck [1], Ricky Granger [1], Erica Jennings [1], Maya Nebbia [1], Andrew Thauwald [1], Greg Lyon [1], Rama K. Maganti [1] and Cara J. Westmark [1,2,*]

[1] Department of Neurology, University of Wisconsin, Madison, WI, 53706, USA; prwestmark@wisc.edu (P.R.W.); tswietlik@wisc.edu (T.J.S.); erunde2@wisc.edu (E.R.); bcorsiga@wisc.edu (B.C.); rnissan@wisc.edu (R.N.); bboeck@wisc.edu (B.B.); rhgranger@wisc.edu (R.G.); emjennings2@wisc.edu (E.J.); mnebbia@wisc.edu (M.N.); thauwald@wisc.edu (A.T.); gregory.lyon@focus.org (G.L.); maganti@neurology.wisc.edu (R.K.M.)

[2] Molecular Environmental Toxicology Center, University of Wisconsin, Madison, WI, 53706, USA

* Correspondence: westmark@wisc.edu; Tel.: +1-608-262-9730

† These authors contributed equally to the work.

Citation: Westmark, P.R.; Swietlik, T.J.; Runde, E.; Corsiga, B.; Nissan, R.; Boeck, B.; Granger, R.; Jennings, E.; Nebbia, M.; Thauwald, A.; et al. Adult Inception of Ketogenic Diet Therapy Increases Sleep during the Dark Cycle in C57BL/6J Wild Type and Fragile X Mice. *Int. J. Mol. Sci.* **2024**, *24*, x. https://doi.org/10.3390/ijms25126679

Academic Editors: Lucia Margari and Emilia Matera

Received: 31 May 2024
Revised: 10 June 2024
Accepted: 14 June 2024
Published: 18 June 2024

Copyright: © 2024 by the authors. Licensee MDPI, Basel, Switzerland. This article is an open access article distributed under the terms and conditions of the Creative Commons Attribution (CC BY) license (https://creativecommons.org/licenses/by/4.0/).

Figure S1

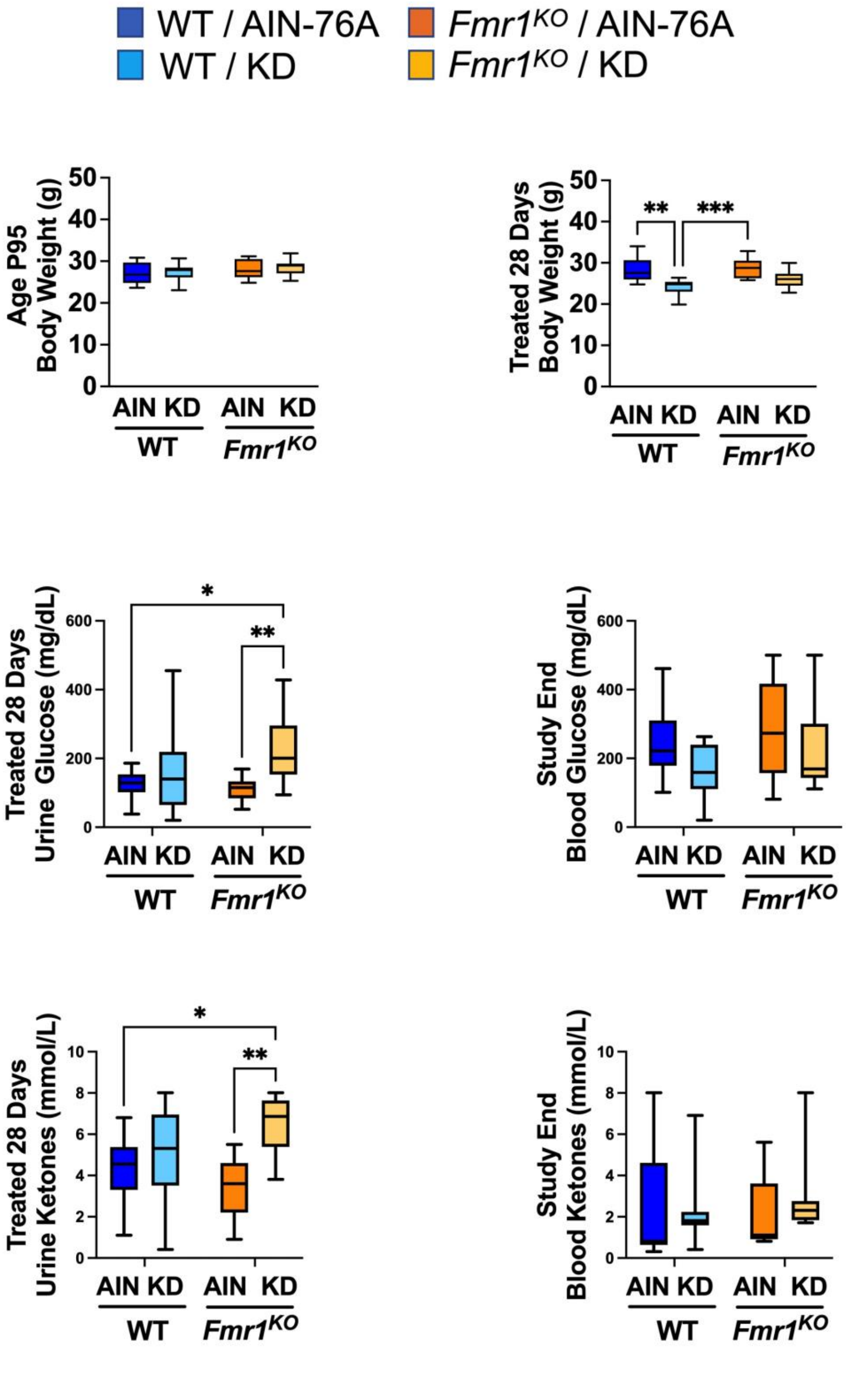

Figure S1. Biometrics as a Function of KD Initiated at P95. Mice were weighed at P95 before KD and 28 days post commencement of KD. Urine glucose and ketone measurements were assessed at 28 days post-KD treatment. Blood glucose and ketone measurements were assessed fasting at euthanization. Statistics with GraphPad Prism included 2-way ANOVA with post-hoc Tukey multiple comparison tests. Asterisks indicate statistical significance where * $p \leq 0.05$, ** $p < 0.01$, *** $p < 0.001$.

Figure S2

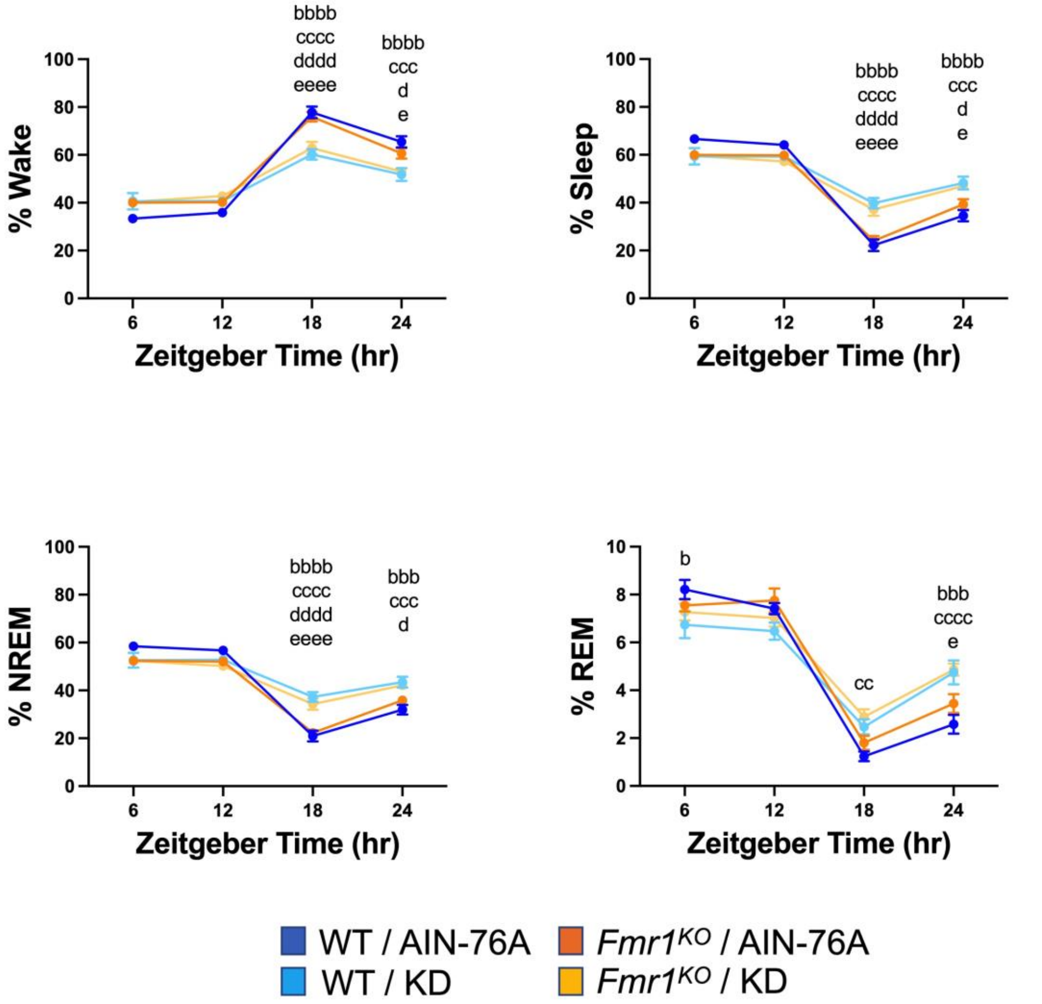

Figure S2. Effect of KD Initiated at P95 on Sleep Architecture in WT and *Fmr1*^{KO} Mice as a Function of Time of Day. EEG/EMG recordings were scored for wake, sleep, NREM and REM activity and the data parsed into 6-hour bins starting at Zeitgeber time zero. The average percent sleep state was plotted versus Zeitgeber time bin for (**A**) % wake, (**B**) % sleep, (**C**) % NREM, and (**D**) % REM. Mouse cohorts included WT fed AIN-76A (n=12), WT fed KD (n=10), *Fmr1*^{KO} fed AIN-76A (n=11), and *Fmr1*^{KO} fed KD (n=12). Statistics with GraphPad Prism included 2-way ANOVA with repeated measures with post-hoc Tukey multiple comparison tests. Key for statistical significance: WT/AIN-76A versus *Fmr1*^{KO}/AIN-76A = ″a″, WT/AIN-76A versus WT/KD = ″b″, WT/AIN-76A versus KO/KD= ″c″, WT/KD versus KO/AIN-76A= ″d″, KO/AIN-76A versus *Fmr1*^{KO}/KD= ″e″, and WT/KD versus *Fmr1*^{KO}/KD= ″f″; and a $p \leq 0.05$, aa $p<0.01$, aaa $p<0.001$, aaaa $p<0.0001$, etc.

Figure S3

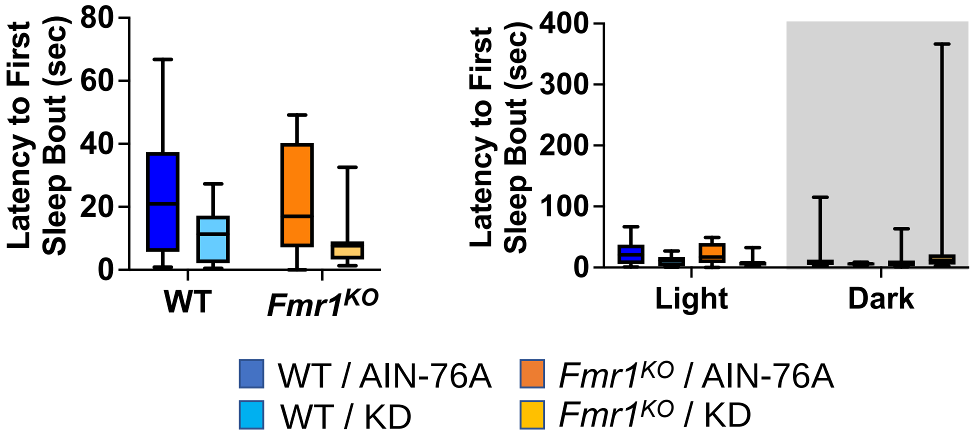

Figure S3. Effect of KD Initiated at P95 on Latency Time to Enter First Sleep Bout in WT and *Fmr1*^{KO} Mice. EEG/EMG recordings were scored for the latency time to the first sleep bout and the data parsed into 24-hour full day and 12-hour light/dark bins starting at Zeitgeber time zero. The average percent latency time was plotted versus Zeitgeber time bin. Statistics with GraphPad Prism included 2-way ANOVA with post-hoc Tukey multiple comparison tests.

Figure S4

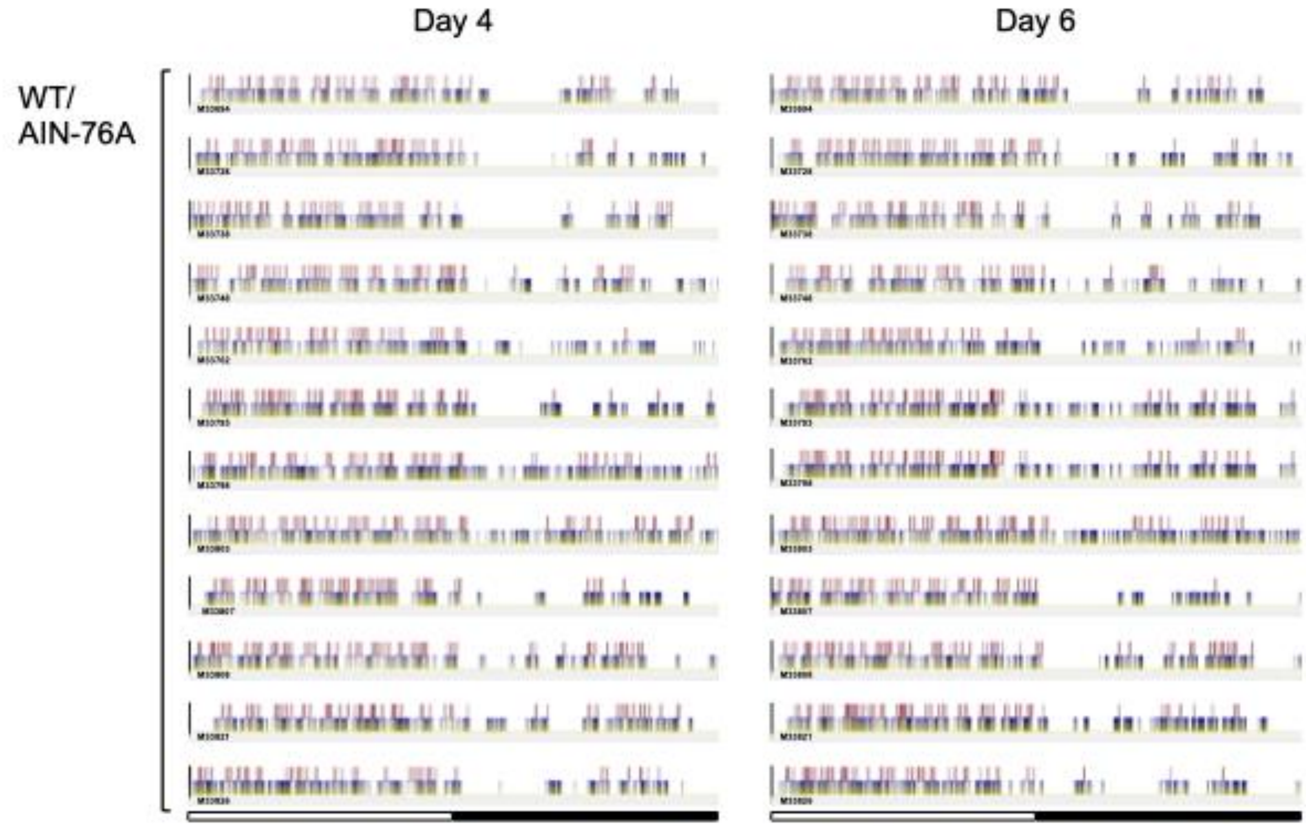

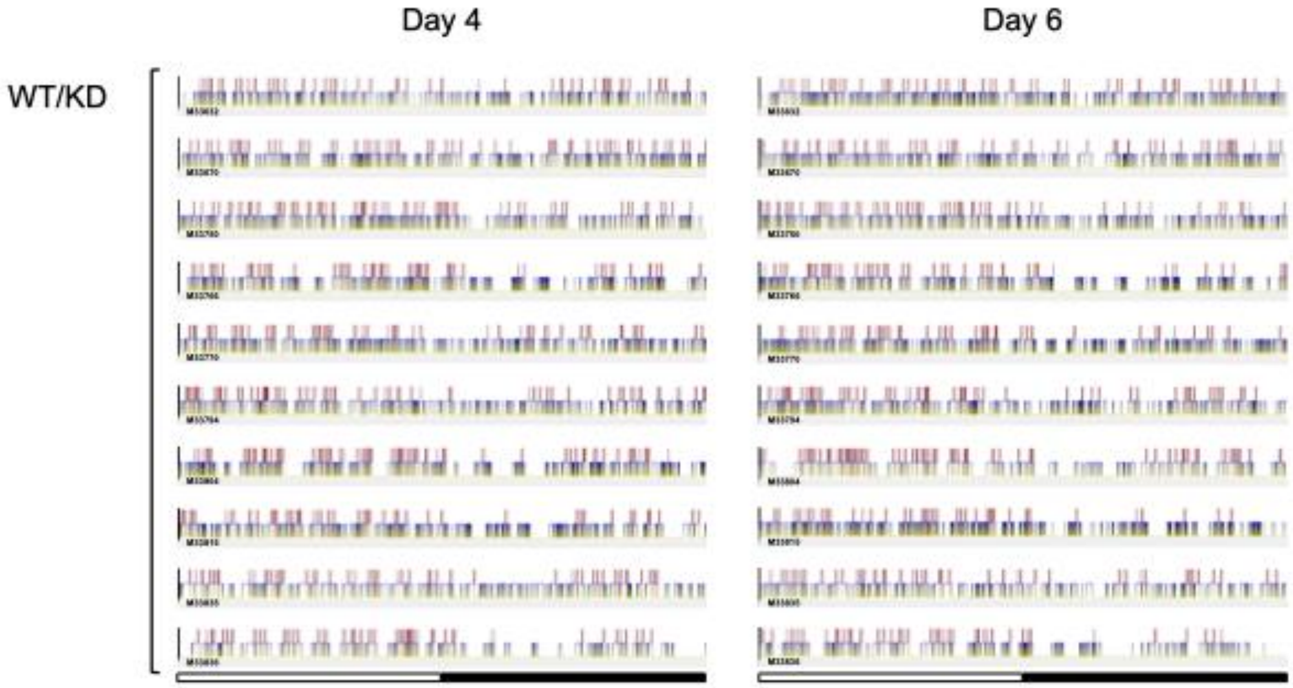
Day 4
Day 6
WT/KD

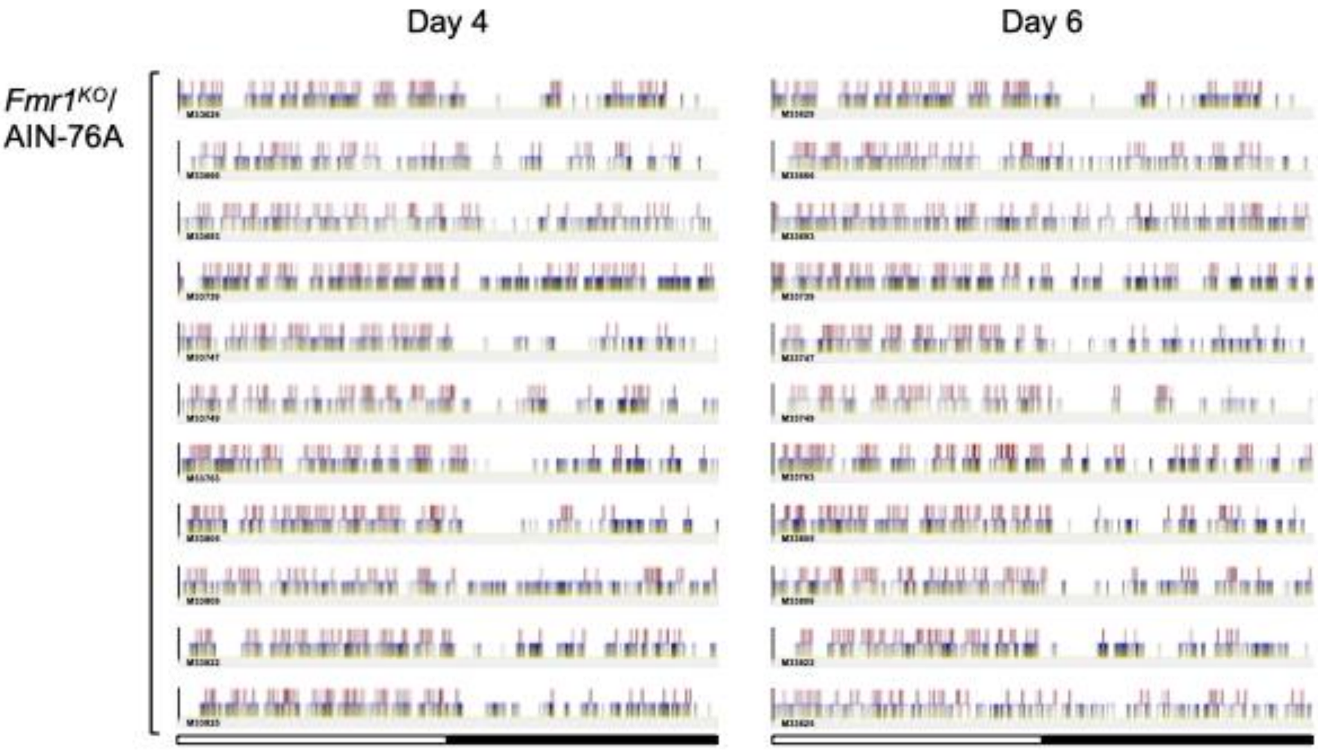
Day 4
Day 6
Fmr1KO/
AIN-76A

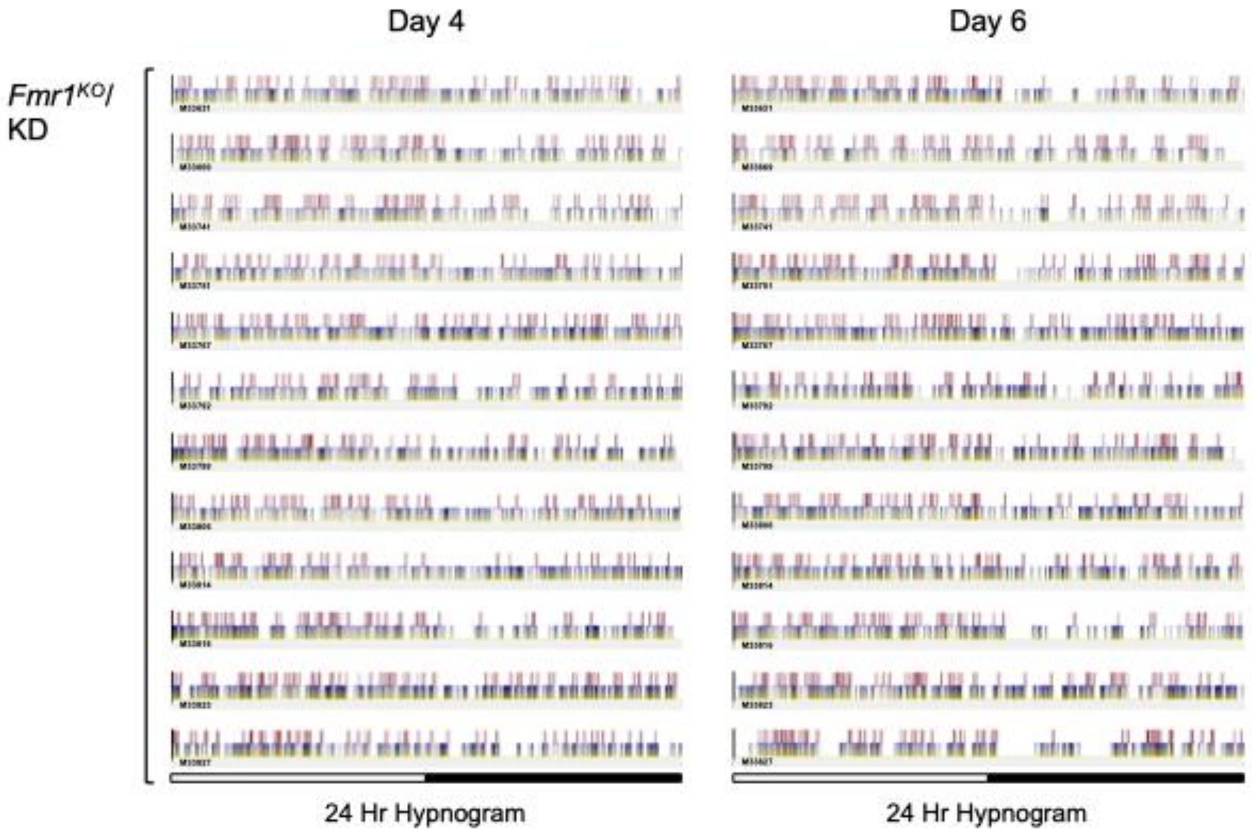

Figure S4. Hypnograms of 24-Hour Sleep-Wake Cycles as a Function of Genotype and Diet. WT and *Fmr1*^{KO} male mice maintained on a 12-hour lights on / 12-lights off cycle were fed AIN-76A versus KD initiated at P95. Hypnograms, which represent sleep stages as a function of time, were generated with Sirenia Sleep® software after manual scoring of sleep states of the EEG/EMG recordings. Mice included WT/AIN-76A (n=12), WT/KD (n=10), *Fmr1*^{KO}/AIN-76A (n=11), and *Fmr1*^{KO}/KD (n=12). The red signal denotes REM sleep, blue signal denotes NREM sleep, and yellow signal denotes wake state.

Disclaimer/Publisher's Note: The statements, opinions and data contained in all publications are solely those of the individual author(s) and contributor(s) and not of MDPI and/or the editor(s). MDPI and/or the editor(s) disclaim responsibility for any injury to people or property resulting from any ideas, methods, instructions or products referred to in the content.
